# An inspired microenvironment of cell replicas to induce stem cells into keratocyte-like dendritic cells for corneal regeneration

**DOI:** 10.1038/s41598-023-42359-9

**Published:** 2023-09-11

**Authors:** Mahsa Fallah Tafti, Hossein Aghamollaei, Mehrdad Moosazadeh Moghaddam, Khosrow Jadidi, Shahab Faghihi

**Affiliations:** 1 Stem Cell and Regenerative Medicine Group, National Institute of Genetic Engineering and Biotechnology, 14965/161, Tehran, Iran; 2 Chemical Injuries Research Center, Systems Biology and Poisonings Institute, Baqiyatallah University of Medical Sciences, Tehran, Iran; 3 Tissue Engineering and Regenerative Medicine Research Center, Baqiyatallah University of Medical Sciences, Tehran, Iran; 4 Vision Health Research Center, Semnan University of Medical Sciences, Semnan, 1435916471 Iran

**Keywords:** Stem-cell differentiation, Biomaterials, Tissue engineering

## Abstract

Corneal stromal disorders due to the loss of keratocytes can affect visual impairment and blindness. Corneal cell therapy is a promising therapeutic strategy for healing corneal tissue or even enhancing corneal function upon advanced disorders, however, the sources of corneal keratocytes are limited for clinical applications. Here, the capacity of cell-imprinted substrates fabricated by molding human keratocyte templates to induce differentiation of human adipose-derived stem cells (hADSCs) into keratocytes, is presented. Keratocytes are isolated from human corneal stroma and grown to transmit their ECM architecture and cell-like topographies to a PDMS substrate. The hADSCs are then seeded on cell-imprinted substrates and their differentiation to keratocytes in DMEM/F12 (with and without chemical factors) are evaluated by real-time PCR and immunocytochemistry. The mesenchymal stem cells grown on patterned substrates present gene and protein expression profiles similar to corneal keratocytes. In contrast, a negligible expression of myofibroblast marker in the hADSCs cultivated on the imprinted substrates, is observed. Microscopic analysis reveals dendritic morphology and ellipsoid nuclei similar to primary keratocytes. Overall, it is demonstrated that biomimetic imprinted substrates would be a sufficient driver to solely direct the stem cell fate toward target cells which is a significant achievement toward corneal regeneration.

## Introduction

Stroma is the thickest corneal tissue layer, mainly consisting of keratocytes and collagen-rich extracellular matrix (ECM)^1^. The arrangement and orientation of collagen fibrils and keratocytes of the ECM have a crucial role in the cornea’s unique properties including mechanical strength, avascularity, shape, and transparency^1, 2^. Keratocytes are the primary quiescent, dendritic/satellite cells that maintain ECM levels in the tissue and regulate the ECM components in the tissue microenvironment^1, 3^. Primary keratocytes are responsible for expressing aldehyde dehydrogenase 3 family, member A1 (ALDH3A1), keratocan, lumican, and cluster of differentiation 34 (CD34) in the corneal stroma. Keratocytes display the morphological characteristics of fibroblasts (flatten and spindle) or myofibroblasts (large and polygonal) which express fibronectin and α smooth muscle actin (α-SMA) in vitro or when removed from stromal tissue^4, 5^.

Corneal stromal diseases are significant causes of vision impairment and eventually blindness. Impaired corneal function in most cases occurs due to incidents like corneal scarring and stromal dystrophies which results in considerable keratocytes loss^6^. Currently, corneal transplantation is the gold method to restore visual function in corneal diseases. However, the lack of enough donor which results in long waiting list as well as corneas graft rejections necessitate alternative treatment strategies^7^. Over the last decade, cell-based therapy to circumvent transplantation issues has been a promising approach to restore the loss of keratocytes^8^. Corneal regeneration requires appropriate biomaterials, cells, and bioactive molecules to create functional tissue replacements. The cells in corneal regeneration can be human ocular keratocytes or corneal stromal stem cells (CSSCs). The isolation of intraocular cells along with limited healthy corneal donors are the limitations for corneal regeneration^5^. Plus, differentiation of non-ocular stem cells into specific corneal cells due to their ease of separation, multipotency, and immunomodulatory properties has been also considered as a significant cell source in regenerative medicine^9^.

To maintain or change the stem cells’ fate under diverse physiological conditions, an instructive multidimensional microenvironment as a biological niche is required^10^. The microenvironment should possess various characteristics including surface chemistry and physical cues that could guide cell behaviors^11^. The topography of ECM is one of the main physical cues that can direct cell adhesion, growth, migration, and differentiation^12^. Thus, it is crucial to employ biofabrication techniques in order to simulate the chemical composition and nanoscale architecture of the ECM under physiological conditions^13^. For instance, substrate topography can strongly affect the polarity of different cell types through cytoskeletal and nucleoskeletal rearrangement^14^. There are also evidences that micro/nano-sized topographies could potentially alters the gene expression and stimulate cytoskeleton formation leading to cell differentiation. In addition, micro/nanotopographies could convert physical cues such as mechanical forces into biochemical responses^15^.

Cell-imprinting, as a novel physical method uses poly dimethyl siloxane (PDMS) to mimic stem cell environment and modulate differentiation and proliferation of the cell. In 2013, the cell-imprinting for induction of stem cell differentiation into target cells was developed^16^. This study acquired micro/nanotopographies of cell-imprinted substrates based on templates of mature and dedifferentiated chondrocytes. The adipose derived-stem cells (ADSCs) were then cultured on these cell-imprinted substrates and guided to obtain the specific shape of the template cell type. The cell imprinting method has been performed for differentiation of variety of multipotent stem cells toward tenogenic^17^, osteogenic^18^, keratinogenic^14^, Schwan^19^, neural^20^, as well as cardiomyocyte differentiation from human induced pluripotent stem cells (iPSCs)^21^. In all studies, changes in cell morphology may be the primary contributors for controlling cell behavior along with genes and proteins expression profile^16–18^. Previous studies have demonstrated that in order to generate corneal ECM, non-ocular adult stem cells such as ADSCs could adopt keratocyte phenotype under specific conditions^8, 22, 23^. The achieved cells have also been considered for bioengineering of corneal tissue and clinical studies^24^.

**Figure 1:**
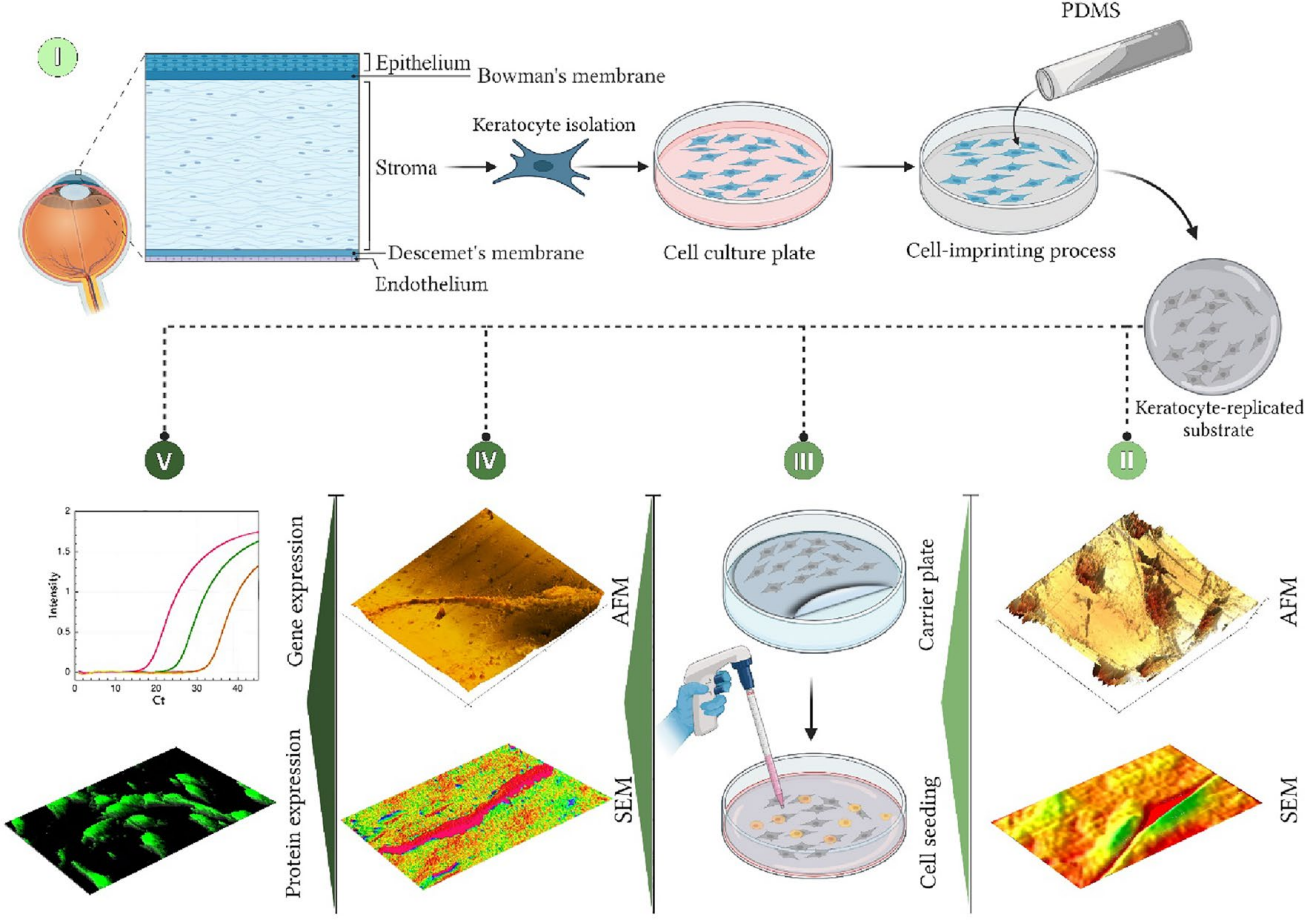
Graphical illustration of (**I**) Isolation and culture of primary corneal stromal cells (keratocytes), to prepare keratocyte-imprinted PDMS substrate, (**II**) characterization of the imprinted substrate by SEM and AFM, (**III**) culture of stem cells on imprinted PDMS, (**IV**) characterization of the cells on the patterned substrate using SEM and AFM, (**V**) evaluations of gene and protein expression by differentiated cells.

Previously, we have indicated the efficacy of a decellularized ECM from corneal stromal tissue together with keratocyte conditioned-medium for inducing differentiation of ADSCs into keratocyte-like cells^25^. The aim of the present study is to evaluate the ability of cell-imprinted substrates for inducing differentiation of ADSCs, mainly to compensate the isolation of healthy keratocytes with a promise to achieve improved efficacy in medical applications. Thus, keratocyte cells were isolated from human corneal stromal and grown to transmit their ECM architecture and cell-like topographies to a PDMS substrate by mold casting. The ADSCs were then seeded on cell-imprinted substrates and their differentiation to keratocytes in two different types of media was evaluated by real-time PCR and immunocytochemistry (Fig. 1). Moreover, the emplaced cells on PDMS were assessed in terms of the cytoskeletal features by actin staining, expression of specific keratocyte marker (keratocan), and nuclei shape in comparison with primary keratocytes and ADSCs. Finally, the nuclei of these cells were analyzed based on particle characteristics including roundness, circularity, and aspect ratio.

## Materials and methods

This research was carried out in accordance with the ethical principles for medical research consisting of human samples as outlined in the Declaration of Helsinki guidelines established by the World Medical Association. Informed consent was obtained from all subjects and/or their legal guardian(s). All procedures including the use of human tissues in this study were approved by the ethical institutional board of the National Institute of Genetic Engineering and Biotechnology (NIGEB), Iran, with the identification number of IR.NIGEB.EC.1399.7.21.B.

### Isolation and culture of keratocytes

Human keratocytes were isolated from healthy corneal stromal tissue of 10 donors with an average of 52 years old (range 34–70). The remained healthy corneal stromal segments were provided during the Descemet stripping endothelial keratoplasty (DSEK) surgery. In this procedure, the endothelium along with Descemet's membrane was removed.

Descemet stripping of the recipient and a posterior donor tissue insertion are prepared with a microkeratome to replace endothelially. In this lamellar surgery, the corneal stromal layer is remained^26^. Remained corneal stromal segments were preserved in Optisol-GS (Bausch & Lomb Surgical, Irvine, California, USA) and transported at 4 °C to the laboratory. The isolation and extraction of corneal stromal cells was performed according to literature by Yam et al. in 2015^5^. Briefly, after washing with sterile phosphate-buffered saline (PBS), the central button (8 mm in diameter) was trephined and treated using resolved dispase II (20 mg/ml; Gibco) diluted in DMEM/F12 (Gibco), 1 h at 37 °C, followed by gentle scraping to eradicate the corneal epithelium. The stromal tissue fragments were digested with collagenase I (1 mg/ml; Gibco) diluted in DMEM/F12, for 12 h at 37 °C. Single cells were obtained after centrifuging at 1400*g* and suspended in a suitable maintenance medium. For cell culture, a keratocyte basal medium (KBM) with some modifications was prepared from Dulbecco's Modified Eagle Medium/F12 Ham's (DMEM/F12; Biosera, France) supplemented with 1% insulin–transferrin–selenate (ITS) (Gibco), 1% minimum essential medium (MEM) nonessential amino acids (Gibco), 1 mM l-ascorbate 2-phosphate (Sigma-Aldrich), and 100 U/ml penicillin and 100 µg/ml streptomycin (Invitrogen). In addition, soluble amnion extract (AME; Life Cell, Iran) and 5% fetal bovine serum (FBS; Gibco) were added to the cells’ medium^5^. Cells were cultured on tissue culture polystyrene (TCP) for one week at 37 °C and 5% CO_2_, and the medium was changed every three days.

### Corneal stromal cells characterization

After 7 days of culture, the cells with high confluency were characterized by optical (Nikon, Japan), and fluorescent microscopy (Olympus IX71, Japan). The quantitative real-time polymerase chain reaction (RT-PCR) and immunocytochemistry (ICC) were also performed. Total RNA was isolated with cell lysis using TRIzol reagent according to the manufacturer’s recommendations (GeneAll, South Korea). Chloroform and isopropanol were added to the TRIzol reagent to separate and precipitate total RNA, respectively. After resolving the RNA in RNase-free distilled water, a NanoDrop (Thermo Fisher Scientific, USA) was used to determine the concentration and purity of the separated RNA.

For cDNA synthesis from the RNA, a SuperScript II reverse transcriptase Kit (GeneAll, South Korea) was employed. An optimized RT-PCR profile including an initial denaturation of 10 min at 95 °C, then 40 cycles of 15 s for denaturation at 95 °C, 45 s for annealing at 60 °C, and 30 s for extension at 72 °C was considered. RT-PCR was done using a direct dye binding (SYBR Green; Applied Biosystems) and Rotor-Gene (Corbett Robotics, Australia). The gene expression was normalized using β-actin. Primer sequences used for RT-PCR are shown in (Table 1-PCR). All the samples were run as triplicates.

**Table 1:** Primer sequences.

Sequence definition	Forward sequence	Reverse sequence
*Lumican*	CCTGGTTGAGCTGGATCTGT	TAGGATAATGGCCCCAGGA
*Keratocan*	ATCTGCAGCACCTTCACCTT	CATTGGAATTGGTGGTTTGA
*ALDH3A1*	CATTGGCACCTGGAACTACC	GGCTTGAGGACCACTGAGTT
*CD34*	CTTGGGCATCACTGGCTATT	TCCACCGTTTTCCGTGTAAT
*ACTA2*	CTATGCCTCTGGACGCACAAC	CAGATCCAGACGCATGATGGCA
*β-actin*	TTCTACAATGAGCTGCGTGTGG	GTGTTGAAGGTCTCAAACATGAT

For protein characterization, a double staining using specific keratocyte markers including keratocan and lumican as well as a single staining using α-SMA as a myofibroblast marker were considered. Briefly, the cells were fixed for 40 min with 4% paraformaldehyde in PBS on ice and permeabilized with 0.5% Triton X-100 in PBS at room temperature for 5 min. The samples were blocked in 1% bovine serum albumin (BSA) (Merck, Darmstadt, Germany) and incubated overnight at 4 °C with primary antibodies including rabbit anti-Keratocan (1:200 dilution, Biozol) and rabbit anti-Lumican antibody, 1:100 dilution, Sigma-Aldrich). After pouring off the antibody solutions and washing the cells with PBS, the staining signals were revealed by the secondary antibodies including fluorescein isothiocyanate (FITC)-conjugated goat antirabbit IgG (1:100; Santa Cruz Biotechnology, USA.) and phycoerythrin (PE)-conjugated goat antimouse IgG (1:100; Santa Cruz Biotechnology, USA). Cell nuclei were counterstained with 4,6-diamidino-2-phenylindole (DAPI) (1 mg/ mL; Santa Cruz Biotechnology, USA) for 5 min.

To examine myofibroblast phenotype, the cells after fixation and permeabilization were diluted by rabbit polyclonal antibody to α-SMA (1:100 dilution, Biorbyt, UK) overnight at 4 °C. After rinsing with PBS, FITC-conjugated goat antirabbit IgG (1:100; Santa Cruz Biotechnology, USA) as a secondary antibody was added in the dark at room temperature for 1 h. Before imaging, the immune-stained cells were stained with DAPI (1 mg/mL; Santa Cruz Biotechnology, USA) for 5 min. The fluorescent signals were visualized by an inverted microscope (Olympus IX71, Japan). The percentages of proteins expression were measured using ImageJ software (http://imagej.nih.gov/ij/; provided in the public domain by the National Institutes of Health, Bethesda, Maryland, USA). The immunostaining process for all the experiments was performed in triplicate and all photographs were taken at 20 × magnification.

### Immunophenotypic characterization of ADSCs

Human adipose tissue-derived stem cells were provided from the stem cell technology research center, Bonyakhteh institute, Tehran, Iran. The cells were cultured in a T-75 flask and were maintained in DMEM/F12 (Gibco) supplemented with 10% FBS (Gibco) and 100 U/ml penicillin and 100 µg/ml streptomycin (Invitrogen) at 37 °C and 5% CO_2_. The medium was replaced every three days. Characterization of the stem cells was performed by optical microscopy and flow cytometry.

For immunophenotypic characterization second passage of ADSCs were used. ADSCs were stained with conjugated antibodies including CD105-PE (eBioscience; USA), CD90-APC (BioLegend; USA), CD73-PE-Cy7 (BioLegend; USA), and CD45-FITC (BioLegend; USA). The cells were then trypsinized and the suspension was centrifuged at 300 g for 4 min. A total of 5 × 10^5^ cells were dissolved in 0.2 ml PBS and incubated with conjugated antibodies for 20 min in a dark room^27^. The samples were analyzed using a flow cytometer (BD FACSVerse, BD Biosciences, USA) for identification of specific fluorescence channels of each antibody.

### Fabrication of cell-imprinted substrates

Fresh corneal stromal cells were grown in 6-well culture plates to obtain high confluency. In order to maintain the details of cells morphology, they were fixed with 4% cold paraformaldehyde (Sigma-Aldrich) in PBS (pH 7.4) for 20 min at room temperature. The silicone-based substrates were fabricated using silicon elastomer kit PDMS (SYLGARD 184, RTV, Dow Corning, USA) according to the manufacturer’s preparation protocol. Combination of the Sylgard 184 polymer and the curing agent were at ratio of 10:1 and poured onto the fixed cells. After incubation at 37 °C for 24–48 h, the PDMS was peeled off from the cells, rinsed in a NaOH solution (1 M) at 100 °C, and autoclaved to remove inactivate biological agents adhered to the substrates. A PDMS without cell imprinting was used as control. The total mass of imprinted PDMS and non-imprinted PDMS, temperature, and curing time for all the samples were similar. The imprinted substrates were examined with optical (Nikon, Japan), scanning electron microscopy (SEM) (Philips XL30, Netherlands), and atomic force microscopy (AFM) (Autoprobe CP-Research, Veeco, USA) equipped with IP 21 software.

### Culture of hADSCs

The hADSCs at a density of 5 × 10^4^ in 100 μL were seeded on the imprinted PDMS, as well as un-patterned PDMS and TCP controls in 6-well culture plates and incubated overnight at 37 °C. The total volume of media was then reached to 2 ml in 5 h and cultured for 1, 2, and 3 weeks with medium exchange every 3 days. The cell culture procedure was similar for both KBM and DMEM/F12 media.

### Microscopic observation

The hADSCs cultured on the keratocyte-imprinted substrate were characterized by SEM and AFM. For SEM, the cells were fixed with 4% fresh paraformaldehyde for 40 min. After rinsing with deionized water, dehydration was performed on each specimen in a series of graded acetone alcohols (Merck Millipore) (50%, 75%, 90% and 100%) for 30 min in each bath. Before imaging, the samples were sputter coated with a thin layer of gold and viewed by SEM (Philips XL30, Netherlands). Seeded cells on patterned PDMS was also imaged by AFM (Autoprobe CP-Research, Veeco, USA) equipped with IP 21 software.

### Differentiation of hADSCs on imprinted substrates

#### RNA extraction and gene expression analyses

ADSCs cultured on patterned PDMS, un-patterned PDMS, and TCP under both media cultures after one, two, and three weeks were analyzed by Real-time PCR similar to primary keratocytes as described in Sect. “Corneal stromal cells characterization”. The primers are listed in Table 1.

#### Immunostaining of cells on PDMS substrates

After two and three weeks, the protein expression of hADSCs cultured on patterned and un-patterned silicon substrates (control) in both types of media was analyzed by double staining of anti-keratocan and anti-lumican as mentioned in Sect. “Corneal stromal cells characterization”.

Moreover, the cultured stem cells on patterned substrates after two and three weeks were examined for myofibroblast phenotype (as mentioned in Sect. “Corneal stromal cells characterization”).

The morphology of cells emplaced on cell-imprinted substrates (DMEM/F12, at day 21), primary keratocytes, and ADSCs were also compared by confocal microscopy. For this purpose, the seeded cells on patterned substrates were detached with trypsin/ EDTA (Sigma-Aldrich) and cultured on slides. After 72 h, the cells were permeabilized and incubated with rabbit anti-Keratocan antibody (1:200 dilution, Biozol) overnight at 4 °C. After washing with PBS, the cells were incubated with FITC-conjugated goat antirabbit IgG (1:100; Santa Cruz Biotechnology, USA) Subsequently, for F-actin staining, rhodamine phalloidin (1:100 dilution; Invitrogen), a high-affinity F-actin probe conjugated to the red–orange fluorescent dye tetramethylrhodamine (TRITC) was added to the samples for 1 h at room temperature in dark. Finally, the cells were washed with PBS and stained with DAPI (1 mg/mL; Santa Cruz Biotechnology, USA) for 5 min. The cells were imaged by confocal microscope (Leica, TCS SP5, Germany). The staining process was performed in triplicate for all the samples and photographs were taken at 40 × magnification.

### Nucleus shape analysis

It has been reported that the shape and orientation of the nucleus changes during the differentiation process of cells. Therefore, the shape properties of nuclei for hADSCs, cultured on patterned PDMS (at day 21), and primary keratocytes were analyzed and compared. For this purpose, 20 nuclei images were taken at 40 × magnification for each sample. The cells were selected randomly and analyzed based on particle parameters with the ‘Analyze Particles’ function within Image J to identify the circularity = 4πArea/perimeter^2^, roundness = 4Area/πmajor axis^2^, and aspect ratio = major axis length of approximate particle/minor axis length of approximate particle^28, 29^.

### Statistical analysis

One-way ANOVA with Bonferroni test was used to compare real-time PCR (normalized only to the housekeeping gene β actin) and protein expression levels. Data analysis was performed in SPSS for default analysis to compare absolute qPCR expression values. For nucleus analysis Kruskal–Wallis test was performed and surface parameters were analyzed using independent *t* test. The data were presented as mean ± standard deviation (SD) and considered significant when *p* < 0.05.

### Ethics approval

The study was confirmed by the Ethics Committee of the National Institute for Genetic Engineering and Biotechnology (NIGEB)** (**IR.NIGEB.EC.1399.7.21.B).

## Results

### Cell characterization

#### Primary keratocytes

**Figure 2:**
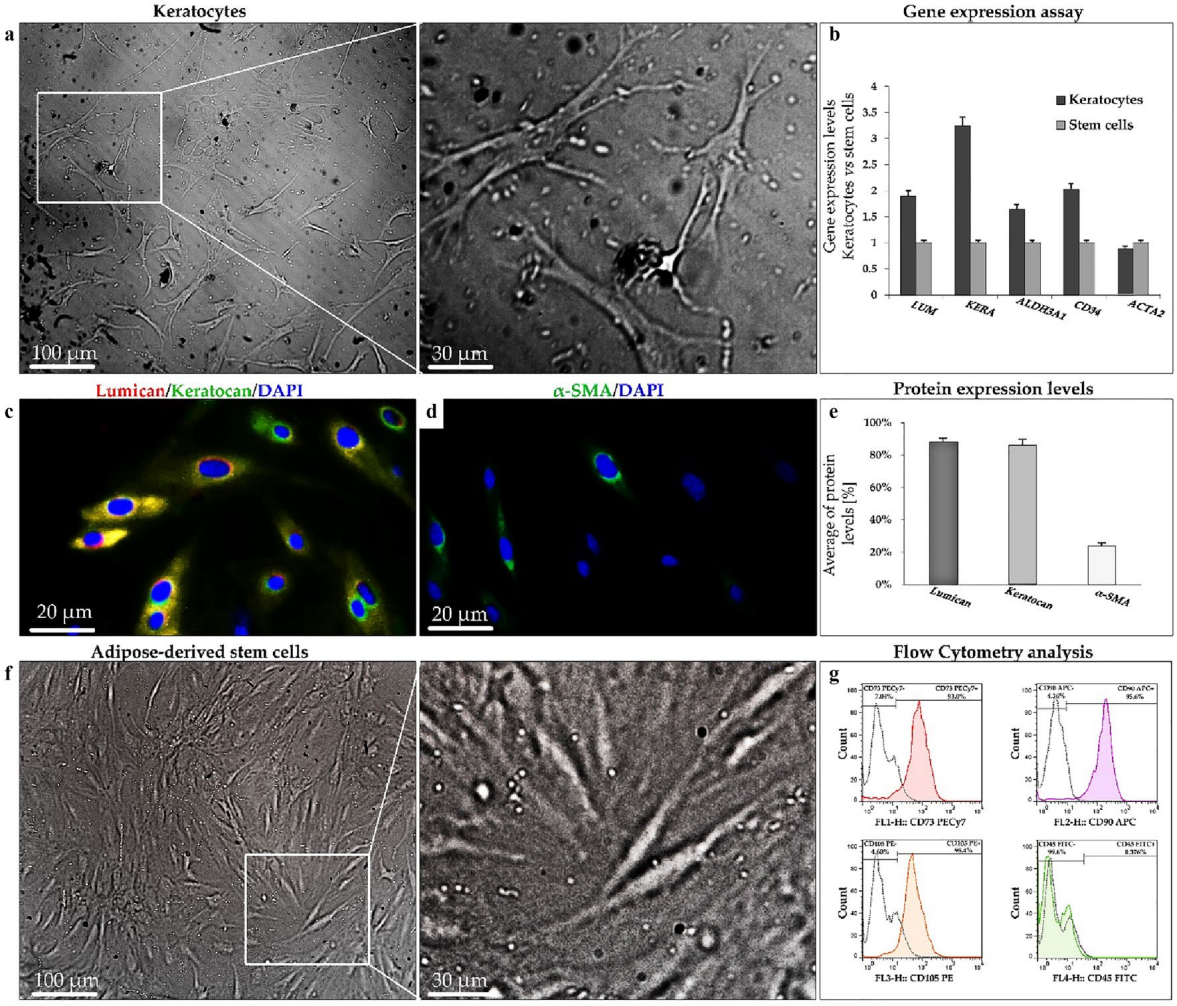
Keratocytes and ADSCs characterization. (**a**) Light microscopy photographs of cultured keratocytes with dendritic morphology. (**b**) The gene expression profile demonstrated higher expression of positive keratocyte markers, including *Lumican* (*LUM*), *Keratocan* (*KERA*), *ALDH3A1*, and *CD34* and minimal expression of myofibroblast marker (*ACTA2*) in keratocytes and ADSCs. (**c**,**d**) Immunofluorescence images of human keratocytes cultured for Keratocan (green)/lumican (red)/nuclei (DAPI, blue), and α-SMA (green)/nuclei (DAPI, blue). (**e**) Representative of average protein levels. (**f**) Light inverted microscopy photographs of ADSCs with spindle shape morphology. (**g**) Flow cytometry results showing up-expression of CD105, CD73, CD90, and down-expression of CD45 in hADSCs.

Three prominent features of human primary corneal stromal cells are dendritic morphology and expression of keratocyte-specific markers including lumican and keratocan. On the contrary, they should have a negligible expression of fibroblasts/myofibroblasts markers (e.g. α-SMA) whereas expression of α-SMA in keratocytes has been observed in vitro^5^. Dendritic morphology of cells was observed and confirmed by optical microscopy (Fig. 2a). The keratocyte-specific markers of the primary cells were also assessed by real-time PCR (Fig. 2b) and immunocytochemistry (Fig. 2c,d) to determine evidence of phenotypic keratocytes. The gene expression assay showed significant expression of specific markers including *Lumican* (*LUM* ≈ 1.8-fold), *Keratocan* (*KERA* ≈ 3.1-fold), *ALDH3A1* (≈ 1.6-fold), *CD34* (≈ twofold) in primary keratocytes as compared to the control group (ADSCs). However, similar to ADSCs, the low-expression of *ACTA2* (α-SMA) gene was observed in primary keratocytes (Fig. 2b). In addition, as can be seen in Fig. 2e, human keratocytes are shown more than 70% immunoreactivity for both lumican and keratocan specific markers and low expression of α-SMA, even in the presence of serum.

#### hADSCs

A spindle-shaped morphology and specific cluster of differentiation (CD markers) on the cell surface are the essential criteria for characterizing ADSCs. Therefore, hADSCs were characterized in terms of morphology and expression of ADSCs-specific surface antigens (e.g. CD105, CD90, and CD73). In flow cytometry, the expression of the MSC-specific surface antigens (e.g. CD105, CD90, and CD73) was higher than 90% of the ADSCs populations while indicated low or zero expression level for hematopoietic markers like CD45^27^. Accordingly, the morphology of ADSCs in Fig. 2f, is exhibited as an elongated spindle-shaped morphology under optical microscopy. The analysis of ADSCs-specific markers for isolated stem cells confirmed the positive percentage of cell population at 95.4% for CD105, 93% for CD73 and 95.6% for CD90, and 0.376% for CD45 (Fig. 2g).

### Characterization of cell-imprinted substrates

**Figure 3:**
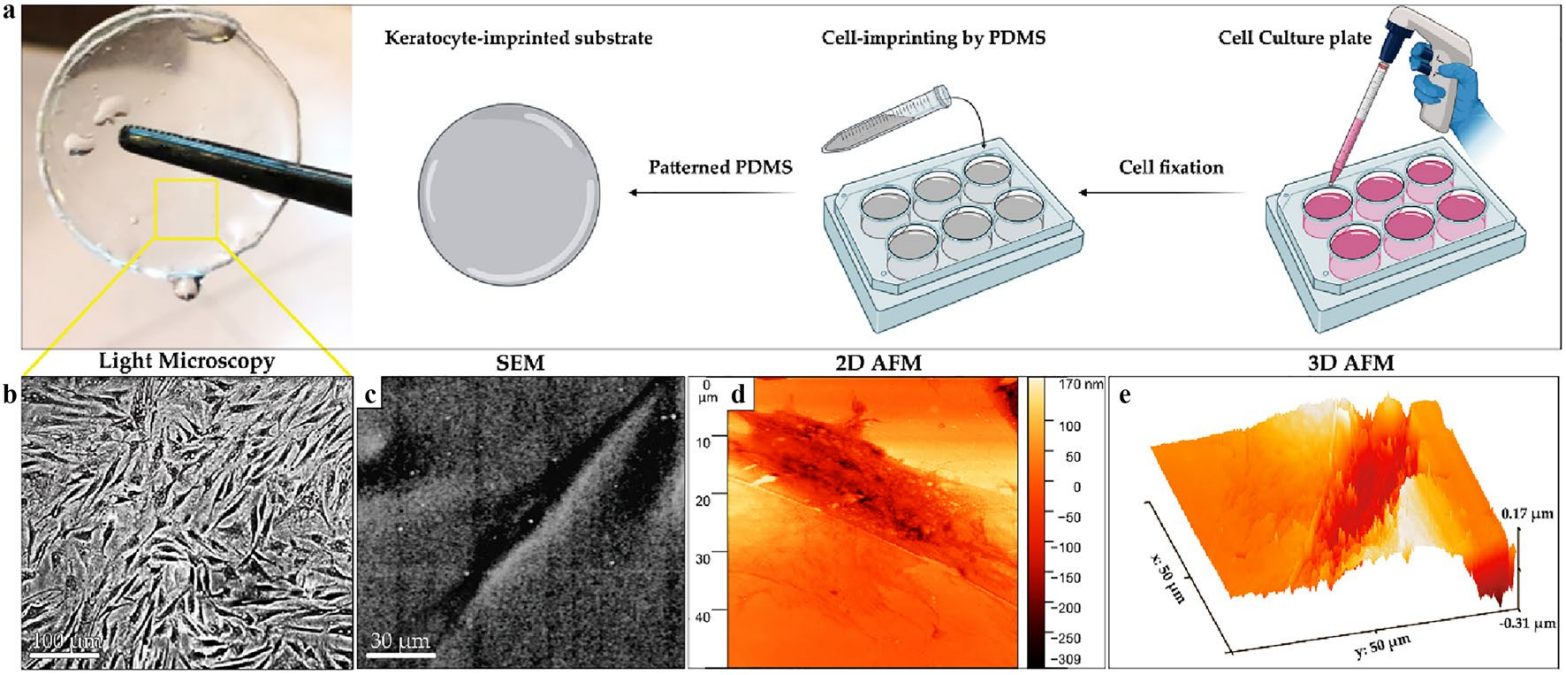
Observation of imprinted substrates by optical, SEM, and AFM. (**a**) Preparation process of patterned PDMS. (**b**) Light image of the cell-imprinted replicas on surface of PDMS. (**c**) The SEM image of keratocyte-imprinted substrate. (**d**,**e**) 2D and 3D AFM images of the patterned substrates.

Biomimetic materials have potential for adjusting the proliferation, self-renewal, and differentiation of stem cells into different lineages^11^. PDMS is a silicone material with soft, flexible, transparent, and biocompatible properties that can flow slowly and pattern the precise topography and geometry of the cell surface at nano-scale during the molding process^14^. As mentioned earlier, keratocyte culture could get associated with fibroblast/myofibroblast-related changes, which reduce the expression of keratocyte-specific markers, the rate of proliferation and ECM formation that limit the use of these cells^5^. To overcome this challenge, the cell imprinting method (Fig. 3a) was performed using primary keratocytes cultivated on polystyrene plates. The PDMS was poured on the confluent cells that have previously were fixed with 4% cold paraformaldehyde. The imprinted PDMS substrates were then characterized with optical microscope, SEM and AFM (Fig. 3). It should be noted that it is very important that imprinted substrates possess a high confluency of fixed cell replicas, therefore, KBM was used to propagate primary keratocytes in a culture plate which is indicated in Fig. 3b. The keratocyte-imprinted substrates were also characterized by SEM (Fig. 3c) and AFM in 2D (Fig. 3d) and 3D views (Fig. 3e).

### Surface roughness analysis of cell-imprinted substrates

**Figure 4:**
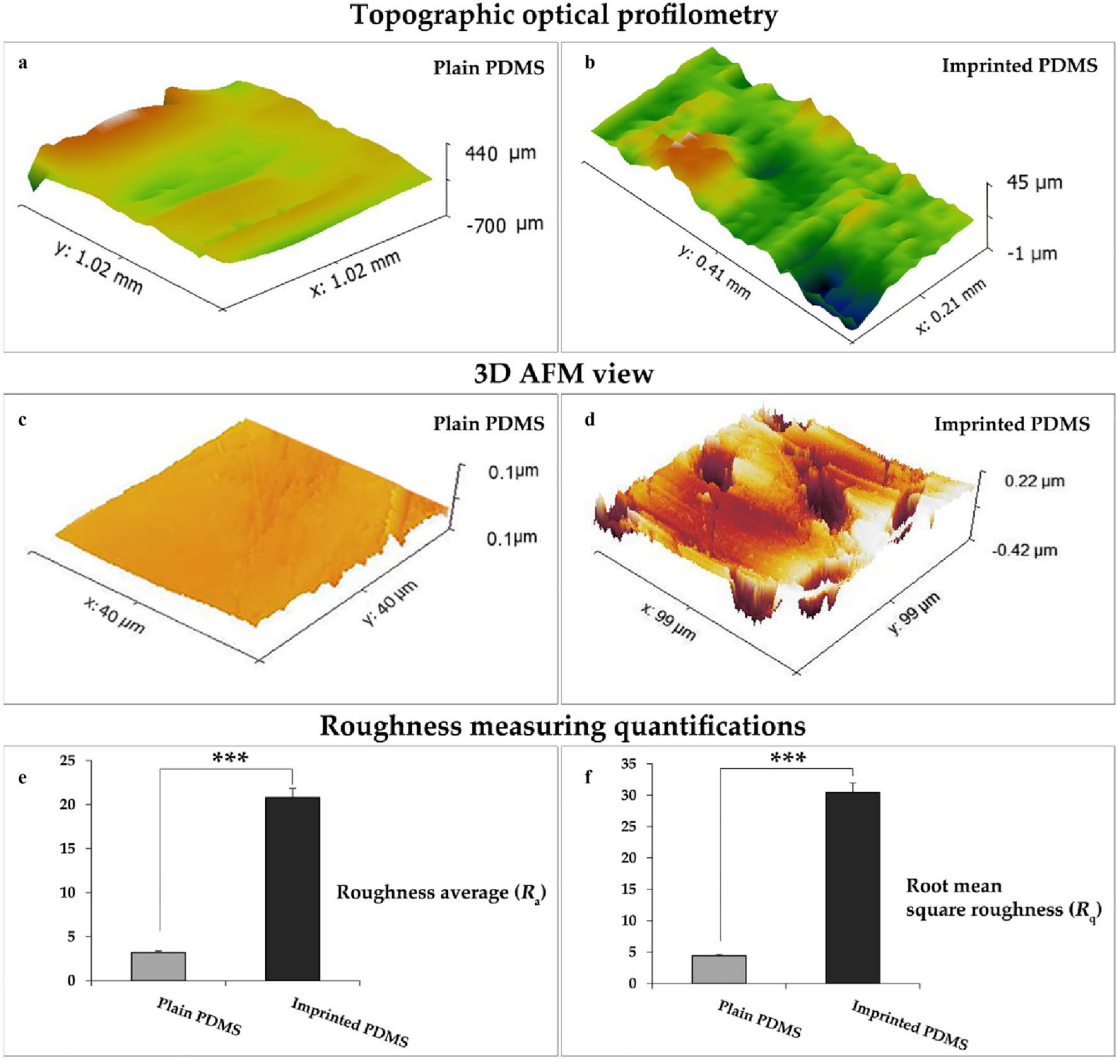
Evaluation of surface roughness of PDMS substrates. (**a**,**b**) Profilometer maps of plain PDMS and patterned substrate. (**c**,**d**) AFM images of plain PDMS and patterned substrate. (**e**,**f**) Statistical analysis of surface parameters that shows significant increase of *R*_a_ and *R*_q_ in patterned substrate as compared to plain substrate. Data are mean ± SD. ***p < 0.001.

Based on Hou Y. et al. study, the increasing surface topographical parameters including roughness average (*R*_*a*_) and root mean square roughness (*R*_q_) can affect the surface roughness during a gradient progression from a flat to rough topography which can affect cell fate^30^. Therefore, the surface topography and roughness of keratocytes imprinted substrates and plain PDMS substrates, were thoroughly imaged by AFM and analyzed by the Gwyddion software. A profilometry map of a plain PDMS (Fig. 4a) compared to a keratocyte-imprinted substrate (Fig. 4b). 3D AFM images of plain PDMS and imprinted PDMS are indicated in Fig. 4c,d. The results showed nanometer amplitude features from the keratocyte-imprinted substrate with the highest level of *R*_*a*_ 28.09 nm and *R*_q_ 42.26 nm. Conversely, the highest levels of *R*_a_ and *R*_q_ for plain substrates of PDMS were 4.9 nm and 5.2 nm, respectively. Surface topography analyses indicated the increased *R*_a_ and *R*_q_ for keratocyte-imprinted substrate in comparison with plain PDMS (Fig. 4e,f).

### SEM and AFM analyses

**Figure 5:**
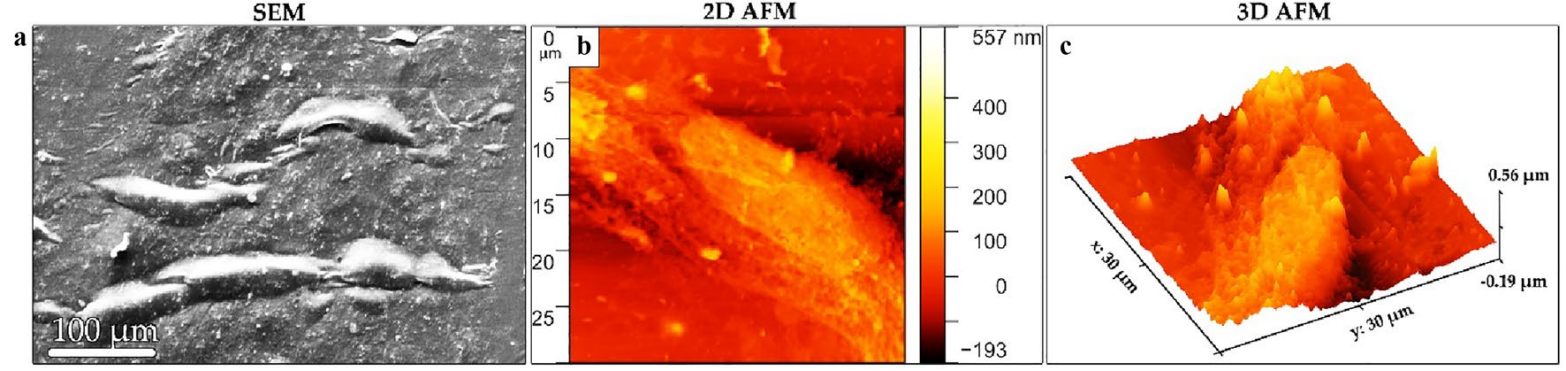
Characterization of cells on patterned substrates. (**a**) SEM image illustrates the ADSCs cultured on keratocytes-imprinted substrate. (**b**) AFM images of the ADSCs cultivated on keratocytes-imprinted substrate are shown in 2D and (**c**) 3D.

In order to confirm the placement of ADSCs on keratocyte-imprinted substrate and patterned shapes, SEM (Fig. 5a) and AFM (Fig. 5b,c) were used. Figure 5b and c show AFM images with high resolution of an emplaced stem cells in the cell pattern cavity in 2D and 3D modes.

### Height profile of the substrates

**Figure 6:**
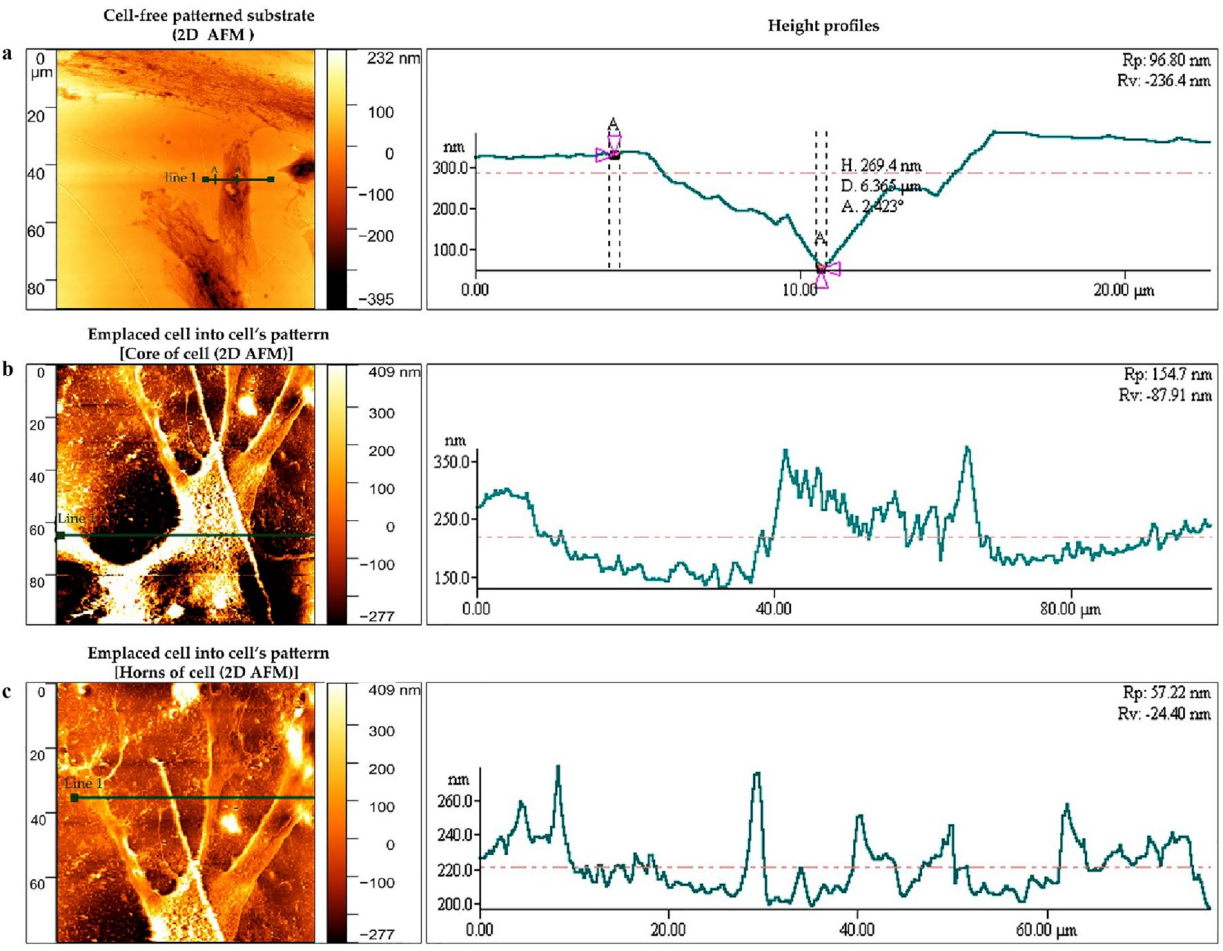
2D AFM topographic images of substrates for height profile. (**a**) A height profile of patterned substrate without cell (between A-A points). (**b**) Height profile after cell seeding on the imprinted substrate. (**c**) an irregulate height profile after emplacing the cell horns on the PDMS surface.

To obtain knowledge about surface topography parameters such as maximum peak to valley height (*R*_p–v_), an imprinted substrate without a cell (Fig. 6a) and an imprinted substrate with seeded cell (Fig. 6b,c) were evaluated with a single line marked on the AFM micrographs. Figure 6a shows *R*_v_ for a patterned substrate without a cell revealing a value of − 236.4 nm that has a higher depth than the substrate with emplaced cell. Conversely, the value of *R*_p_ for the free-patterned substrate (Fig. 6a) was 96.80 nm compared to 154.7 nm for the substrate with cell (Fig. 6b). A different height profile can be seen in Fig. 6c with increasing and decreasing *R*_p_ values, suggesting emplacing cell horns on the substrate’s surface.

### Differentiation analysis

**Figure 7:**
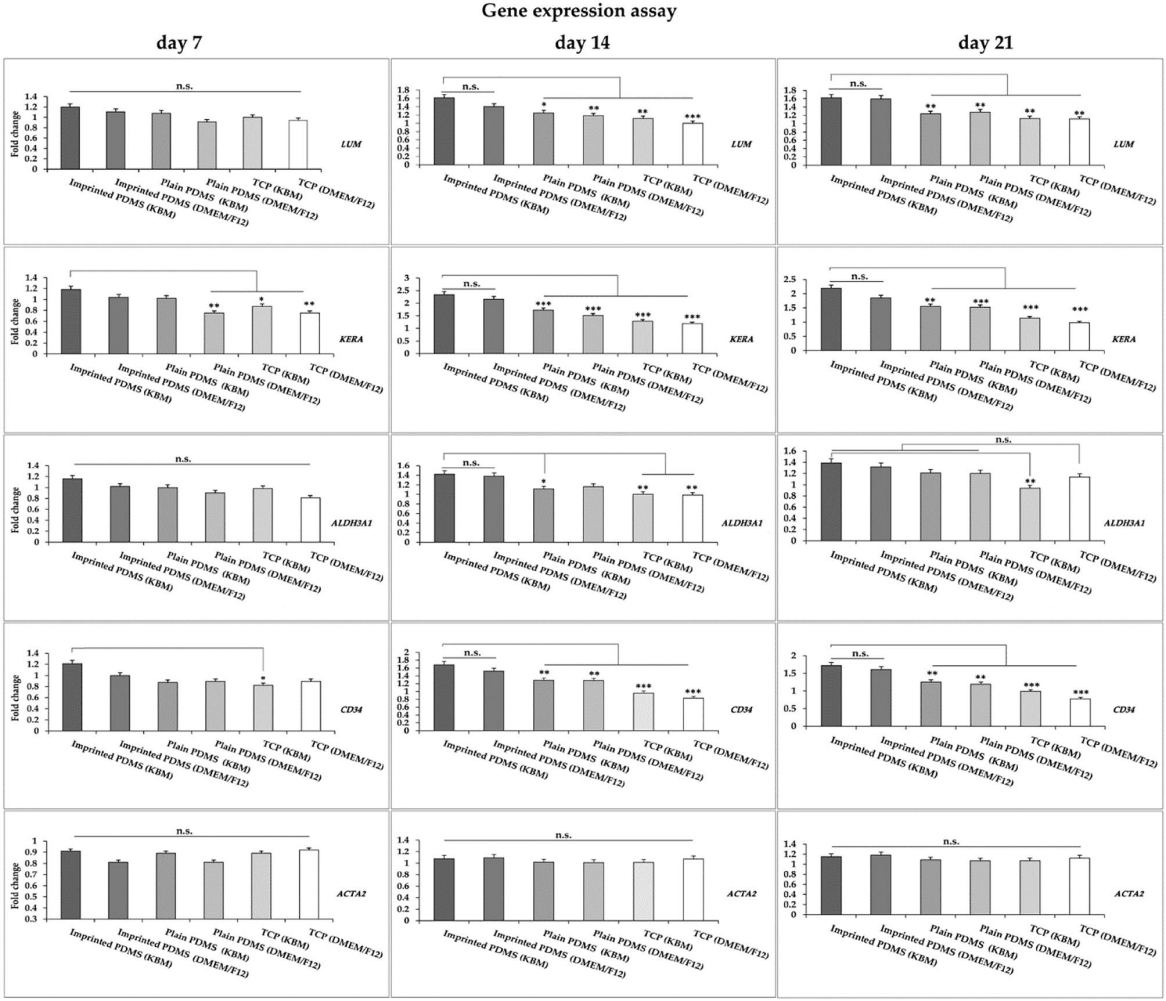
Quantitative analysis of gene expression by ADSCs after 7, 14 and 21 days of culture on cell-imprinted substrate compared to the plain PDMS and TCP as controls. Data are mean ± SD. *p < 0.05, **p < 0.01, ***p < 0.001. *ns* non significant.

The expression of key keratocyte genes after 7, 14 and 21 days of cell culture on cell-imprinted substrates using KBM and DMEM/F12 was performed (Fig. 7). The plain PDMS substrate and TCP were used as control groups. Plain PDMS was used to understand the contribution of PDMS for ADSCs differentiation regardless of the surface features.

#### Real-time PCR

Figure 7 presents the expression of *Lumican (LUN)*, *Keratocan (KERA)*, *ALDH3A1*, and *CD34* as keratocyte-specific genes, and also *ACTA2 (α-SMA)* relative to the housekeeping gene β-actin. A significant increase of *KERA* expression was observed in ADSCs after 1 week of culture on patterned PDMS (KBM) as compared to control substrate, plain PDMS (DMEM/F12, *p* = 0.006), TCP (KBM, *p* = 0.035), and TCP (DMEM/F12, *p* = 0.004). As for the *CD34* gene, an increased expression was observed for ADSCs cultured on patterned PDMS (KBM) compared to TCP (KBM, *p* = 0.027).

After 14 days of culture, a significant increase in expression of *LUM* gene was observed in the cells cultured on keratocyte-imprinted substrate (KBM) in comparison to controls, plain PDMS (KBM, *p* = 0.011), plain PDMS (DMEM/F12, *p* = 0.003), TCP (KBM *p* = 0.001), and TCP (DMEM/F12, *p* = 0.000). Similar result was detected in expression level of *KERA* in ADSCs cultivated on patterned PDMS (KBM) compared to controls (plain PDMS, TCP (KBM/DMEM/F12)) (*p* = 0.000). The expression of *ALDH3A1* gene was also significantly higher on patterned PDMS (KBM) as compared to those cultured on plain PDMS (KBM, *p* = 0.022), TCP (KBM, *p* = 0.002), and TCP (DMEM/F12, *p* = 0.001) whereas the expression level of *ALDH3A1* on plain PDMS (DMEM/F12) was not significant. As for *CD34*, there was a significantly higher expression level in ADSCs cultivated on patterned PDMS (KBM) compared to plain PDMS substrates (KBM and DMEM/F12, *p* = 0.005) and TCP substrates (KBM and DMEM/F12, *p* = 0.000). Increased expression of these genes was not statistically significant for the ADSCs cultured on patterned PDMS (KBM) in comparison with patterned PDMS (DMEM/F12). In addition, expression of *ACTA2* gene by the ADSCs cultivated on TCP and plain PDMS (controls) substrates was similar to the patterned PDMS substrates (KBM and DMEM/F12).

After 21 days of culture, the expression of *LUM* gene was remained significantly higher in the ADSCs cultivated on patterned PDMS (KBM) compared to controls, including plain PDMS (KBM, *p* = 0.004), plain PDMS (DMEM/F12, *p* = 0.003), TCP substrates (KBM and DMEM/F12, *p* = 0.002). Similarly, the level of *KERA* gene was also higher in the ADSCs cultivated on patterned PDMS (KBM) than control substrates including plain PDMS (KBM, *p* = 0.001), plain PDMS (DMEM/F12), TCP (KBM, DMEM/F12, *p* = 0.000). The expression level of *ALDH3A1* gene was higher in cells cultured on patterned PDMS in comparison to TCP (KBM, *p* = 0.004) while its expression on the other control substrates was not significant. A significant increase of *CD34* gene expression was observed in the ADSCs cultivated on patterned PDMS (KBM) as compared to those cultured on plain PDMS (KBM, *p* = 0.002), plain PDMS (DMEM/F12, *p* = 0.001), and TCP (KBM and DMEM/F12) (*p* = 0.000). Interestingly, the expression of these genes (i.e. *LUM*, *KERA*, *ALDH3A1*, *CD34)* were not significantly different between the ADSCs cultured on patterned PDMS (KBM) and patterned PDMS (DMEM/F12). Also, expression of *ACTA2* gene by the ADSCs cultivated on TCP and plain PDMS (controls) substrates was similar to the patterned PDMS substrates (KBM and DMEM/F12).

The expression of key genes (e.g. *LUM, KERA, ALDH3A1, CD34*) of cultivated ADSCs on imprinted PDMS (DMEM/F12) in compression with plain substrates (KBM and DMEM/F12) after 14 and 21 days are presented in (Tables S1 and S2), respectively.

#### Immunostaining

**Figure 8:**
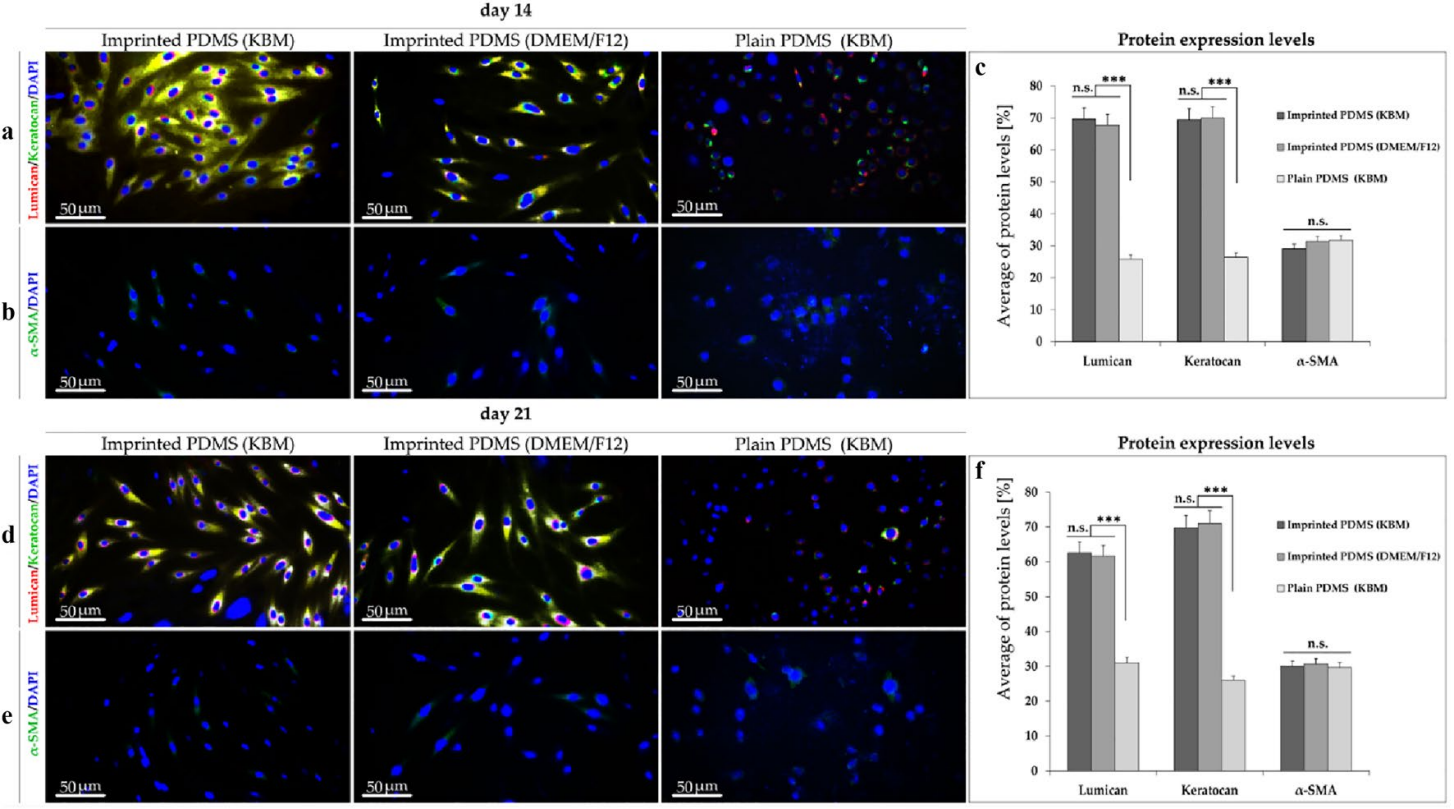
Immunocytochemistry of seeded cells for protein expression after 2 and 3 weeks’ cultivation on patterned PDMS (KBM), patterned PDMS (DMEM/F12) and plain PDMS (KBM) at 40 X magnification. (**a**,**d**) Double staining for lumican (red) and keratocan (green) in ADSCs cultivated on patterned PDMS (KBM), patterned PDMS (DMEM/F12), and plain PDMS (KBM) after 14 and 21-days. (**b**,**e**) Expression of α-SMA protein (green) in the ADSCs on patterned PDMS (KBM and DMEM/F12), and plain PDMS (KBM). (**c**,**f**) Statistical analyses for cultured ADSCs on the imprinted substrates (KBM and DMEM/F12), compared to plain PDMS (KBM) at day 14 and 21, respectively. Data are mean ± SD. ***p < 0.001. *ns* nonsignificant.

Figure 8 shows the immunofluorescent staining of keratocan (green)/lumican (red) and single staining of α-SMA (green) as a myofibroblast marker after 14 and 21 days of culture. After both time points, over 60% of ADSCs cultured on patterned PDMS whether in KBM or DMEM/F12 indicated much higher level of expressing keratocan and lumican compared to plain PDMS (KBM). Statistical analyses show a significantly higher expression of lumican and keratocan proteins on patterned PDMS substrates (KBM and DMEM/F12) rather than plain PDMS (KBM) (*p* = 0.000) at day 14 (Fig. 8c) and day 21 (*p* = 0.000) (Fig. 8f).

On the other hand, the percentage of α-SMA protein expression, 14 and 21 days after cultivation of ADSCs on patterned PDMS (KBM and DMEM/F12) compared to plain PDMS (KBM) were not significantly different. Respecting the expression of lumican and keratocan proteins was well supported by the transcriptional results (Fig. 7); in this evaluation, the ability of ADSCs cultured patterned PDMS (KBM and DMEM/F12) with the ADSCs cultivated on plain PDMS (KBM) were also compared after 14-/21 days. The collected data of the average protein levels demonstrate that emplaced ADSCs on the patterned substrates are not statistically significant according to two-time points (14 and 21 days) (Fig. 8c,f). Herein, for verifying the significance of physical cues on the surface patterned PDMS substrates (KBM and DMEM/F12), we selected plain PDMS (KBM) as the control substrate because of the chemical/inducible factors which are considered for maintaining the keratocyte phenotype.

As indicated, physical cues can alter molecular levels that there might indeed be certain independence of cellular programs, such that inducing a keratocyte phenotype does not necessarily imply shutting down pro-fibroblastic gene expression, particularly in the presence of serum.

### Cell morphology

Keratocytes are dendritic, with an expanded cellular network and compact cell body, enabling them to construct a three-dimensional network of interconnected cells^1^. Conversely, ADSCs are large, flat, elongated (spindle-shaped) cells^31^. The findings of this study showed that after the time considered for differentiation by cell imprinting method, the induced cells show the expected characteristics of a keratocyte cell. However, since cell-imprinted substrates were used for the differentiation of ADSCs based on mechanotranductions, the question arises whether cell differentiation using this method is stable and the cells could maintain their specific phenotype.

**Figure 9:**
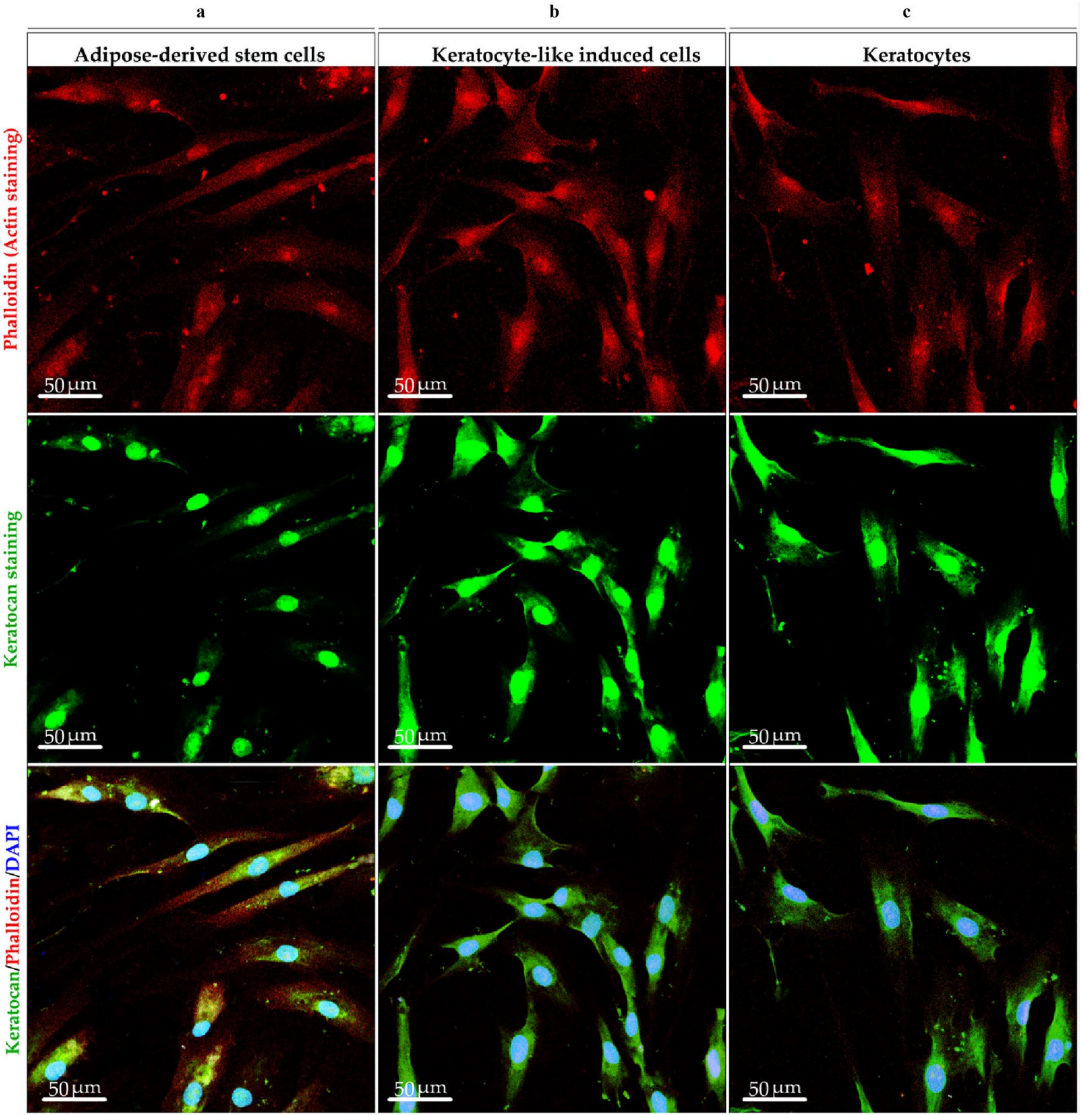
Confocal microscopy of keratocyte-like induced cells compared to ADSCs and primary keratocytes at 40 X magnification. (**a**) Representative confocal images of ADSCs, (**b**) keratocyte like induced cells, and (**c**) primary keratocytes which were double stained by phalloidin (red) and keratocan (green). The DAPI stained cells nuclei (blue) that indicated the changes in the morphology of the ADSCs from long spindle to dendritic morphology in keratocyte-like induced cells and primary keratocytes. Notable expression of keratocan protein is observed in (**b**) keratocyte-like induced cells and (**c**) primary keratocytes.

To address this question, ADSCs on the patterned substrate (DMEM/F12) after three weeks (21 days) were evaluated after detachment and re-culture on a slide to evaluate the keratocyte morphology by F-actin staining and a keratocyte marker expression (Fig. 9) as well as their nuclei (Fig. 9). Similarly, the recorded AFM imaging (Fig. 6b,c) proved the dendritic morphology of seeded ADSCs onto the patterned PDMS (Fig. 9b). The modification of keratocyte-like induced cells with dendritic morphology (Fig. 9b) in compression with ADSCs with elongated spindle morphology (Fig. 9a) is indicated. In the latter case, there are apparent changes in keratocan marker expression and cell morphology.

### Nucleus shape analysis

**Figure 10:**
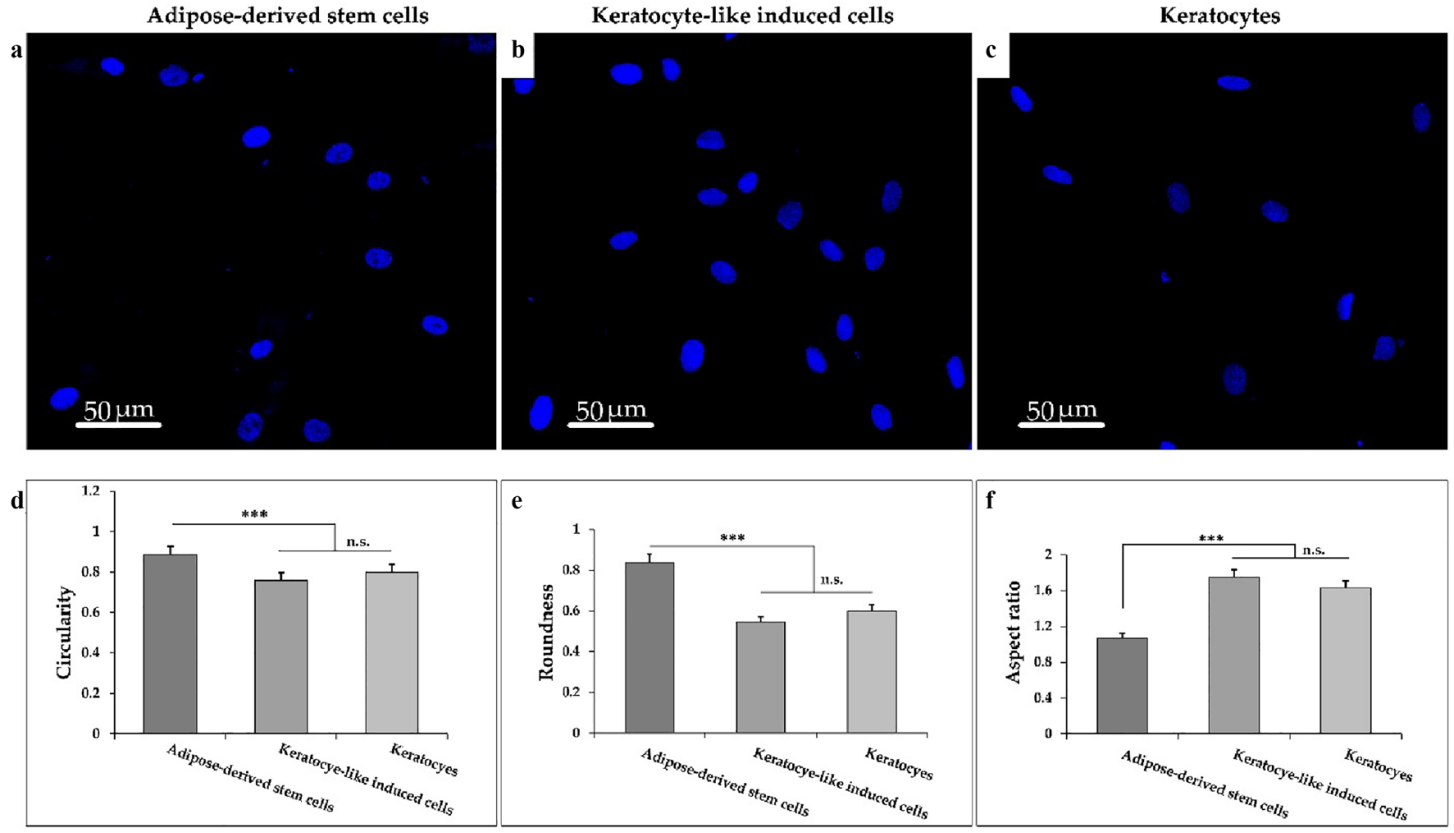
Confocal images of nuclei shape of ADSCs, primary keratocyte, keratocyte-like induced cells at 40X. (**a**) The nucleus images of ADSCs, (**b**) keratocyte-like induced cells and (**c**) primary keratocytes. (**d**–**f**) The nucleus changes of cells by (**d**) circularity, (**e**) roundness, and (**f**) aspect ratio. Statistical analysis by Kruskal–Wallis test indicated significantly higher values for circularity and roundness of the stem cell nuclei than keratocyte and keratocyte-like induced cells. In contrast, significant elongation is illustrated in the keratocyte-like induced cell nuclei compared to ADSCs. Data are mean ± SD. ***p < 0.001. *ns* non significant.

To understand the regulation of mechanotransduction through nanotopographical cues in stem cell differentiation, three parameters in the nuclei shape including circularity, roundness, and aspect ratio of ADSCs, keratocyte-like induced cells, and primary keratocytes were examined (Fig. 10). Figure 10a–c presents the analyses by confocal microscopy for three types of cells. A significant increase of circularity and roundness was calculated for ADSCs compared to primary keratocytes and keratocyte-like induced cells. Conversely, an increased aspect ratio was measured for keratocyte-like induced cells and keratocytes with more ellipsoid shape than ADSCs nuclei with spheroidal morphology.

## Discussion

In this study, the potential of a keratocyte-imprinted substrate combined with a chemical medium (KBM) was evaluated for the differentiation of ADSCs into keratocytes. Obtaining keratocyte-like induced cells with increased keratocyte-specific markers and dendritic morphology was the main outcome of this examination. Many inducible growth factors and bioactive molecules as biological cues for the differentiation of stem cells have been studied, however, cell-derived stem cells cultured in a keratocyte differentiation medium in terms of gene expression levels and cell morphology differ from native keratocytes^9, 32^.

It is recognized that designing substrates with micro-/nano scales could mimic the features of ECM and physical cues as fine-tuning moderators to guide stem cell fate^33, 34^. These ECM properties can influence cellular behavior such as proliferation and differentiation via mechanotransduction pathways^15^. During physical differentiation, cytoskeletal reorganization is activated by inducers like micro-/nano topography surface features and cells geometry which are related to activation of mechanical cues, formation of mature focal adhesion complex, protein kinases, actin filament assembly, nucleus deformation, and finally gene regulation of stem cells^33, 35^. Meanwhile, the exact reason of transmitting extracellular topography to intracellular biological processes is still indistinct.

Imprinted-substrate topographies are usually induced cell differentiation by sharing signaling transduction pathways with chemical stimuli^36^. Although there are many similarities between physical and chemical differentiation, it is recognized that induced differentiation by substrates have a different profile as compared to chemical stimuli used in media culture^33^. The present study is the first report on whole cell-imprinting method to direct differentiation of ADSCs into keratocytes based on substrates of imprinted keratocytes-like topographies. To culture of propagated primary keratocytes and culturing ADSCs on keratocyte-patterned substrate, we used KBM which prepared from effective factors including ITS, l-ascorbate 2-phosphate, and AME. ITS in the presence of insulin provides a stimulatory role in cell proliferation along with maintaining keratocyte markers and dendritic morphology^37^. L-ascorbate 2-phosphate is a nontoxic phosphate derivative of ascorbic acid and can be used as a differentiation chemical factor to induce stem cell differentiation into a keratocyte lineage^38^. The presence of l-ascorbate 2-phosphate to increase collagen and expression of keratan sulfate proteoglycans (lumican and keratocan) in keratocyte medium is also effective^39^. AME is similar to amniotic membrane with high level of growth factors that prevent inflammation, angiogenesis, scarring, and avoiding the fibroblast transition^40^. Therefore, KBM as a supporting medium was used to maintain the phenotype of cultured keratocytes while preserving dendritic morphology and the expression level of specific markers in proliferated primary keratocytes. Our examinations confirmed the efficacy of KBM in culture of primary keratocytes similar to native phenotypic features such as dendritic morphology and high-gene expression level of markers including keratan sulfate proteoglycans (keratocan, lumican), crystallins (e.g. aldehyde dehydrogenases), and CD34^1^. In contrast, we observed a very low expression of myofibroblast marker (*ACTA2* gene or α-SMA). Therefore, KBM can be used as the chemical medium for the cultivation of primary keratocytes which could play a role to promote the proliferation of keratocyte cells without changing the phenotype of the cells. It should be noted that preserved dendritic morphology of keratocytes is crucial for cell imprinting.

In gene expression analysis, ADSCs cultured on patterned substrates (KBM and DMEM/F12) indicated a significantly enhanced expression of specific keratocyte gene markers as compared with plain PDMS and TCP substrates (KBM and DMEM/F12). ADSCs cultured on patterned PDMS (KBM and DMEM/F12) and plain PDMS (KBM) were also examined based on protein expression levels of keratocan and lumican. The results show positive staining for lumican and keratocan with low expression level of α-SMA. It is evidenced that the induced physical cues of both patterned substrates (KBM and DMEM/F12) not only is effective for maintaining the high expression of keratocan and lumican, but also can play a role in controlling the expression of myofibroblast marker even in the presence of KBM.

The most abundant keratan sulfate proteoglycans in the corneal stroma are lumican and keratocan which are members of the small leucine-rich protein family and are necessary to maintain the transparency and shape. Despite lumican as a glycosylated protein which is present in many tissues, keratocan is a proteoglycan exclusively found in cornea^41, 42^. Keratocan and lumican interact with collagen fibrils and adjust their size and spacing^43^. This is distinguished that within the corneal stroma the keratocytes are anchored by interactions between neighboring keratocytes and the surrounding ECM^44, 45^. CD34 is also an adhesion molecule on corneal keratocytes that keep the keratocytes anchored in their microenvironmental niche between the collagen lamellae^44^. There are large amounts of corneal crystallins in native keratocytes phenotype including aldehyde dehydrogenase that protect the cornea from UV radiation. Also, increased opacity of corneal stroma can be related to significant decrease of crystallins in wound-healing keratocytes^46^. In our gene expression analysis, increased expression of ALDH3A1 gene was specified in ADSCs cultivated on patterned substrate (KBM and DMEM/F12) as compared with controls specially TCP substrates (KBM and DMEM/F12). Moreover, increased expression of keratocyte markers were not significant in stem cells cultivated on patterned substrates either in DMEM/F12 or KBM. Therefore, it is believed that physical cues alone or in combination with inducible factors encourage differentiation of ADSCs into keratocyte-like induced cells.

According to the real-time PCR and ICC results, the keratocyte-patterned substrates (KBM and DMEM/F12) proved to increase keratocyte-specific markers expression. Moreover, the stability of the keratocyte-like induced cells which cultured on a plain substrate without inducible factors was an interesting outcome. The stable fate of keratocyte-like induced cells inside the patterns was examined by trypsinization and re-culture on a bare substrate to assess the dendritic morphology and keratocan expression. The results show keratocyte-patterned substrate could preserve keratocyte-like induced cells nature indicated by keratocan expression and presenting dendritic morphology. It seems the cell imprinting method which inducing physical signals for differentiation in an irreversible method.

It is known that the functional properties of surfaces, including adhesion, hydrophobicity, and biological response of the cells are affected by surface roughness^47^. Despite, understanding the general effects of surface roughness from nano to microscale^48^, how topography-induced signals regulate cell manners is not fully understood^30^. Topography is the roughness of the surface, which is related to the structure of the outermost layer of the surface and is defined by measuring the quality of the ridges or depressions of the surface^49^. It is reported that the increase of *R*_a_ and *R*_q_, a gradient progression from a flat to rough topography, affect the cell fate through the process of mechanotransduction^30^. The keratocyte-patterned substrates indicated significantly increased roughness (*R*a-*R*q) compared to the plain PDMS substrates, which could drive the cell fate through manipulating physicochemical signaling pathways^49^. These processes can be reason of reduced expression of keratocyte proteins in ADSCs cultivated on plain PDMS with low roughness compared to the patterned PDMS.

On the other hand, the topology and stiffness of the ECM are affected by the composition and cross-linking of matrix proteins such as collagen, fibronectin, and elastin. One or more mechanosensors can initiate various downstream signaling pathways in response to mechanical stimuli, allowing the cell to respond appropriately. Therefore, cell behaviors can be influenced by various physical characteristics such as stiffness or topology of the substrate^50^. The topology of the substrate is influenced by dynamics and organization of the actomyosin cytoskeleton^51^ whereas stiffness can be attributed more toward the mechanical characteristics of the substrates and impact on cell behaviors^52–54^. Generally, mechanical stresses, which result in alterations in cell density and morphology, have the ability to impact the mechanical properties of the cell skeleton. Additionally, these forces can govern the activities of the Yes-associated protein (YAP) and transcriptional coactivator with PDZ-binding motif (TAZ) mechanical sensors, leading to changes in cellular behavior and function^35^. Therefore, activation of key nuclear factors (YAP/TAZ) would result in mechanosensing of the ECM that can rearrange the nucleoskeletal structure and regulate the cell morphology. Consequently, increased expression of keratocyte genes and proteins in ADSCs cultured on patterned substrates can be due to activated nuclear transcription factors such as YAP/TAZ followed by focal adhesive complexes, Rho GTPase and actomyosin activities from the cellular skeleton. Here, both imprinted and non-imprinted PDMS substrates were prepared according to Wang et al. having comparable total mass at a ratio of 10:1, with the hypothesis that these PDMS substrates would exhibit similar stiffnesses^55^. Conversely, previous studies have shown that inactivation of YAP/TAZ proteins is usually related to small adhesive areas and is occurred in the presence of a soft matrix of the cells. Moreover, the presence of a soft ECM is correlated to the weakening of the innate tensile forces that inhibit the kinase factors such as ROCK leading to inactivation of YAP/TAZ proteins^35, 56–58^. Since, the mechanical cues are highly associated with translocation of YAP/TAZ (nuclear or cytoplasm), changes in the adhesion sites and nucleus deformation are crucial to modulate stem cell fate^35^.

In this study, the deformation of the nucleus of trypsinized ADSCs induced by physical conditions was analyzed based on important parameters including roundness, circularity, and elongation compared to the ADSCs and primary keratocytes. Several studies have indicated the various effects of intra- and extracellular forces on the nuclear shape based on alterations of cell signaling and gene transcription^14, 59^. Mashinchian et al. assessed differentiation of stem cells cultured on keratinocyte-imprinted substrates, probe the nuclei shape and geometry and chain arrangement of simulated chromatin fibers^14^. Their results demonstrated nucleus deformation of the stem cells from primary spherical to ellipsoidal and secondary spherical could regulate target genes expression. This is in agreement with our results indicating deformation of ADSCs nuclei from round shape to spindle which is similar to primary keratocytes^60^.

The corneal stroma is a dome-shaped tissue with a highly ordered environment consisting of a unique arrangement of collagens and keratocytes^61^. Thinning of stromal tissue is correlated to the loss of keratocytes that produce transparent ECM which are crucial for vison^6^. Meanwhile, ocular stem cells such as CSSCs may have a possible advantage for differentiation into keratocytes^62^. The regeneration of corneal stroma using propagated primary keratocytes under a chemical medium might lead to a transformed phenotype^5^.

Cell-imprinting method that induce ADSCs into keratocyte-like cells could open a wide range of opportunities for corneal stroma regeneration. Even though, cell-imprinting as a shape-dependent differentiation method is considered a seemingly simple method, there are many challenges regarding size, structure, fragility and fluidity of the cells which require immense attention and contemplation^36^. However, creating a biomimetic microenvironment by cell-imprinting method would be a game-changer to circumvent many limitations of chemical factors used in current differentiation methods.

## Conclusion

In summary, cell-imprinted substrates with ECM architecture and cell-like topographies have proven their potency to encourage cell attachment, phenotype switch, and guiding ADSCs' commitment to keratocyte lineage. The expression of keratocyte-specific markers after 14 and 21 days were shown significant differences in the cultivated ADSCs on keratocyte-imprinted substrates (KBM and DMEM/F12) in compression with those cultured on the plain substrates. The cell morphology screening and nucleus analyses for keratocyte-like induced cells demonstrated their native properties. It is indicated that not only topography-related signaling pathways of keratocyte-imprinted substrates are adequate for deriving ADSCs differentiation into keratocytes, but also, they can be overcome common obstacles derived from chemical factors in cell culture media. Therefore, the results of the current study introduce an inexpensive, reliable*,* repeatable differentiation method without using chemical factors. Finally, the cell-imprinting method introduced here, can be considered a promising method for corneal regeneration by using autologous stem cells in clinical applications.

### Supplementary Information

Supplementary Tables.

## Data Availability

The data that support the findings of this study are available from the corresponding author upon request.
