# Supplementary material for: An inspired microenvironment of cell replicas to induce stem cells into keratocyte-like dendritic cells for corneal regeneration
Source: Sci Rep. 2023 Sep 11;13:15012. doi: 10.1038/s41598-023-42359-9 (PMC10495344; doi:10.1038/s41598-023-42359-9)
Supplement: Supplementary file 1 — Supplementary Tables. [file 41598_2023_42359_MOESM1_ESM.docx]

**Table S1: Multiple compression of qPCR analysis at day 14**

| Dependent Variable | Group | Groups | Sig. |
| --- | --- | --- | --- |
| *LUMICAN* (*LUM*) | Imprinted PDMS substrate (DMEM/F12) | Imprinted PDMS substrate (KBM) | .348 |
|  |  | Plain PDMS (KBM) | 1.000 |
|  |  | Plain PDMS (DMEM/F12) | .257 |
|  |  | TCP (KBM) | .056 |
|  |  | TCP (DMEM/F12) | .005 |
| *KERATOCAN* (*KERA*) | Imprinted PDMS substrate (DMEM/F12) | Imprinted PDMS substrate (KBM) | .516 |
|  |  | Plain PDMS (KBM) | .001 |
|  |  | Plain PDMS (DMEM/F12) | .000 |
|  |  | TCP (KBM) | .000 |
|  |  | TCP (DMEM/F12) | .000 |
| *ALDH3A1* | Imprinted PDMS substrate (DMEM/F12) | Imprinted PDMS substrate (KBM) | 1.000 |
|  |  | Plain PDMS (KBM) | .058 |
|  |  | Plain PDMS (DMEM/F12) | .202 |
|  |  | TCP (KBM) | .004 |
|  |  | TCP (DMEM/F12) | .003 |
| *CD34* | Imprinted PDMS substrate (DMEM/F12) | Imprinted PDMS substrate (KBM) | 1.000 |
|  |  | Plain PDMS (KBM) | .186 |
|  |  | Plain PDMS (DMEM/F12) | .172 |
|  |  | TCP (KBM) | .000 |
|  |  | TCP (DMEM/F12) | .000 |
| *ACTA2* | Imprinted PDMS substrate (DMEM/F12) | Imprinted PDMS substrate (KBM) | 1.000 |
|  |  | Plain PDMS (KBM) | .306 |
|  |  | Plain PDMS (DMEM/F12) | .249 |
|  |  | TCP (KBM) | .276 |
|  |  | TCP (DMEM/F12) | 1.000 |

**Table S2: Multiple compression of qPCR analysis at day 21**

| Dependent Variable | Group | Groups | Sig. |
| --- | --- | --- | --- |
| *LUMICAN* (*LUM*) | Imprinted PDMS substrate (DMEM/F12) | Imprinted PDMS substrate (KBM) | 1.000 |
|  |  | Plain PDMS (KBM) | .005 |
|  |  | Plain PDMS (DMEM/F12) | .004 |
|  |  | TCP (KBM) | .004 |
|  |  | TCP (DMEM/F12) | .003 |
| *KERATOCAN* (*KERA*) | Imprinted PDMS substrate (DMEM/F12) | Imprinted PDMS substrate (KBM) | .089 |
|  |  | Plain PDMS (KBM) | .217 |
|  |  | Plain PDMS (DMEM/F12) | .143 |
|  |  | TCP (KBM) | .000 |
|  |  | TCP (DMEM/F12) | .000 |
| *ALDH3A1* | Imprinted PDMS substrate (DMEM/F12) | Imprinted PDMS substrate (KBM) | 1.000 |
|  |  | Plain PDMS (KBM) | 1.000 |
|  |  | Plain PDMS (DMEM/F12) | 1.000 |
|  |  | TCP (KBM) | .021 |
|  |  | TCP (DMEM/F12) | .478 |
| *CD34* | Imprinted PDMS substrate (DMEM/F12) | Imprinted PDMS substrate (KBM) | 1.000 |
|  |  | Plain PDMS (KBM) | .012 |
|  |  | Plain PDMS (DMEM/F12) | .005 |
|  |  | TCP (KBM) | .000 |
|  |  | TCP (DMEM/F12) | .000 |
| *ACTA2* | Imprinted PDMS substrate (DMEM/F12) | Imprinted PDMS substrate (KBM) | 1.000 |
|  |  | Plain PDMS (KBM) | 1.000 |
|  |  | Plain PDMS (DMEM/F12) | .356 |
|  |  | TCP (KBM) | .798 |
|  |  | TCP (DMEM/F12) | 1.000 |
